# Resilience in perinatal HIV+ adolescents in South Africa

**DOI:** 10.1080/09540121.2016.1176676

**Published:** 2016-07-08

**Authors:** Arvin Bhana, Claude A. Mellins, Latoya Small, Danielle F. Nestadt, Cheng-Shiun Leu, Inge Petersen, Sphindile Machanyangwa, Mary McKay

**Affiliations:** ^a^ SA Medical Research Council, Health Systems Research Unit, Durban, South Africa; ^b^ School of Applied Human Sciences, University of KwaZulu-Natal, Durban, South Africa; ^c^ HIV Center for Clinical and Behavioral Studies, New York State Psychiatric Institute and Columbia University, New York, NY, USA; ^d^ School of Social Work, The University of North Carolina at Chapel Hill, NC, USA; ^e^ Silver School of Social Work, New York University, New York, NY, USA

**Keywords:** Resilience, HIV+ adolescents, social action theory, mental health

## Abstract

Increasing numbers of perinatally HIV (PHIV+)-infected youth are surviving into adulthood with better access to treatment. However, few studies examine positive outcomes in the face of adversity (resilience) for PHIV+ youth. Social Action Theory (SAT) provided the theoretical framework for this study of PHIV + youth in South Africa (SA), allowing examination of contextual, social, and self-regulatory factors that influence behavioral health. Data were from youth and caregiver baseline interviews, simply pooled from a pilot (N=66) and larger (n=111) randomized control trial (RCT) of the VUKA Family program. For this analysis, outcomes included emotional and behavioral functioning (total difficulties), and prosocial behaviors. Potential SAT correlates included socio-demographics; caregiver health and mental health; parent**-**child relationship factors; stigma, and child coping, support; and self-esteem. Regression analyses adjusted for age, gender, and study revealed significant associations at the contextual, social, and self-regulation level. Lower total child difficulties scores were associated with lower caregiver depression (β = 3.906,p < .001), less caregiver-reported communication about difficult issues (β = 1.882, p = .009) and higher youth self-esteem (β = **-**0.119, p = .020). Greater prosocial behaviors were associated with greater caregiver-reported communication (β = 0.722, p = .020) and child use of wishful thinking for coping (β = 5.532, p = .009). Less youth depression was associated with higher caregiver education (β =**−**0.399, p = .010), greater caregiver supervision (β = **−**1.261, p = .012), more social support seeking (β = **−**0.453, p = .002), higher youth self-esteem (β = **−**0.067, p < .001), lower internalized stigma (β = 0.608, p = .040), and child use of resignation for coping (β = 1.152, p = .041). Our data support evidence-based family interventions that also promote youth self-regulation skills to enhance the health and mental health of PHIV+ youth.

With the increase in the availability of antiretroviral treatment (ART) in South Africa (SA), a large cohort of perinatally HIV (PHIV+)-infected youth, once not expected to outlive childhood, are now aging into adolescence and young adulthood (Sohn & Hazra, 2013; UNAIDS, 2014). However, a set of complex issues related to identification, care, and treatment of children with perinatal HIV infection persists. For example, despite the decrease in the number of children with severe illness at ART initiation, 62% of children were still initiating therapy with advanced disease in 2013 (World Health Organization Stage III/IV disease) (Davies et al., 2013).

In addition to the normative stressors associated with adolescent physiological, psychological, and social development, PHIV+ youth are likely to confront a number of additional challenges, both HIV and non-HIV related, including illness and death of parents and siblings, caregiving responsibilities for younger siblings or other family members, stigma and discrimination related to HIV or orphanhood, and an uncertain future (Lowenthal et al., 2014; Petersen et al., 2010). Studies from SA and other parts of the world have consistently shown that the majority of PHIV+ youth come from families that are larger and poorer, have lower rates of employment, and are living in impoverished communities (Bachmann & Booysen, 2003; Cluver, Boyes, Orkin, & Sherr, 2013). Moreover, high rates of emotional and behavioral health problems among PHIV+ youth have been noted in a number of studies in both high- and low-resource contexts (Bomba et al., 2010; Mellins & Malee, 2013; Mellins et al., 2012; Puthanakit & Siberry, 2013). Various studies from the USA and sub-Saharan Africa have found that PHIV+ youth, similar to same age peers, are engaging in substance use and sexual behaviors, and use condoms inconsistently (Bauermeister, Elkington, Robbins, Kang, & Mellins, 2012; Birungi, Mugisha, Obare, & Nyombi, 2009; Mellins et al., 2011; Tassiopoulos et al., 2013). Furthermore, PHIV+ adolescents and young adults have lower levels of medication adherence and higher levels of drug resistance relative to younger children and adults (Van Dyke et al., 2016), increasing risk of adverse medical outcomes and transmission of HIV to others.

However, it is also true that despite the increased risk and vulnerabilities facing PHIV+ youth, some studies show that many are presenting with behavioral health problems that are the same or less than uninfected peers. For example, several studies have found relatively low rates of substance use (Elkington, Bauermeister, Brackis-Cott, Dolezal, & Mellins, 2009; Mellins, 2013; Mellins & Malee, 2013; Williams et al., 2010) and late onset of sexual behaviors (Bauermeister et al., 2012; Mellins & Malee, 2013) among PHIV+ youth relative to uninfected youth. Although some studies suggest that PHIV+ youth experience high rates of psychiatric disorders and mental health problems, a number of studies have also found no significant differences in rates of psychiatric disorders between PHIV+ and demographically similar uninfected youth, including PHIV-exposed, but uninfected youth and HIV− youth who have HIV+ family members (Gadow et al., 2010; Gadow et al., 2012; Mellins et al., 2012; Sopena, Evangeli, Dodge, & Melvin, 2010). Furthermore, HIV-associated neurocognitive disorders range widely from 8 to 60% in untreated children, suggesting that not all children and adolescents experience the same level of vulnerability (Nassen et al., 2014). Even in studies that have found higher rates of mental health and other problems among PHIV+ youth, large proportions of youth have displayed no risk outcomes (Kapetanovic et al., 2011; Mellins, 2013; Mellins et al., 2011). All this suggests the presence of factors in the lives of some PHIV+ youth that promote resilience, broadly defined as the achievement of positive outcomes despite circumstances that are linked with negative outcomes (Zolkoski & Bullock, 2012).

While few studies specifically related to resilience among PHIV+ youth exist, particularly in low-resource contexts, a review of the evidence from other fields and preliminary work in HIV suggests that the child’s health status and cognitive function, parental health and mental health, as well as stressful life events and disruptive neighborhoods, may be associated with worse mental health outcomes, while parental involvement and communication with the child and parent and teacher social support are associated with positive outcomes (Catalano, Hawkins, Berglund, Pollard, & Arthur, 2002; Gadow et al., 2012; Kia-Keating, Dowdy, Morgan, & Noam, 2011; Luthar, Sawyer, & Brown, 2006; Mellins & Malee, 2013; Petersen et al., 2010). Resilience is, therefore, both a function of characteristics of the individual child and the relationships that support the child, as well as the quality of that child’s environment, which provides the resources necessary for positive development, despite adverse circumstances (Ungar et al., 2007).

The existing literature has consistently emphasized that resilience is most strongly bolstered when protective factors at the levels of individual, family, and community are strengthened (Benzies & Mychasiuk, 2009; Stein et al., 2014). While understanding the areas of risk is essential to targeting these vulnerabilities, these findings do not tell us “how” to intervene. Understanding factors that can facilitate the health and well-being of PHIV+ youth as they age, that is, studying the influences that can bolster resilience, is increasingly valued in designing programs and identifying points of intervention that might most effectively promote positive outcomes and avert negative effects (Mofenson & Cotton, 2013).

As a model of behavior change, Social Action Theory (SAT; Ewart, 1991) offers a useful lens for examining the contextual, social, and individual factors that may explain resilience. SAT emphasizes the context in which behavior occurs and the developmentally driven self-regulatory and social interaction processes that affect adaptive behavior. Contextual elements that have been found to be protective in general child and youth populations include safe neighborhoods, access to quality schools and health services, a stable and sufficient family income, and community financial support (Benzies & Mychasiuk, 2009). At the level of social interactions, parent–child and other family relationship factors have been associated with positive outcomes across populations. For example, parent–child supervision, positive emotional expression, family cohesion, and supportive parent–child communication have been associated with positive youth development (Fergus & Zimmerman, 2005; Zolkoski & Bullock, 2012) and may be critical for PHIV+ youth (Mellins & Malee, 2013). Finally, self-regulatory factors, including self-esteem, coping, and self-efficacy have also been found to be important predictors of resilience (Zolkoski & Bullock, 2012).

Using SAT, the present study explored what (i) contextual, (ii) social regulation, and (iii) self-regulation variables were associated with positive mental health outcomes in a population of young South African PHIV+ adolescents. The SAT model has been previously adapted and used in several studies of youth and adults infected and affected by HIV in the USA (Mellins et al., 2008; Traube, Dukay, Kaaya, Reyes, & Mellins, 2010). More specifically, using our adapted SAT model (see Figure 1), we hypothesize that behavioral health outcomes, including mental health, are influenced by context (e.g. socioeconomic status and caregiver health status), self-regulation (self-esteem, perceived illness stigma), and social regulation (e.g. family communication and supervision and social support) factors.

**Figure 1.**
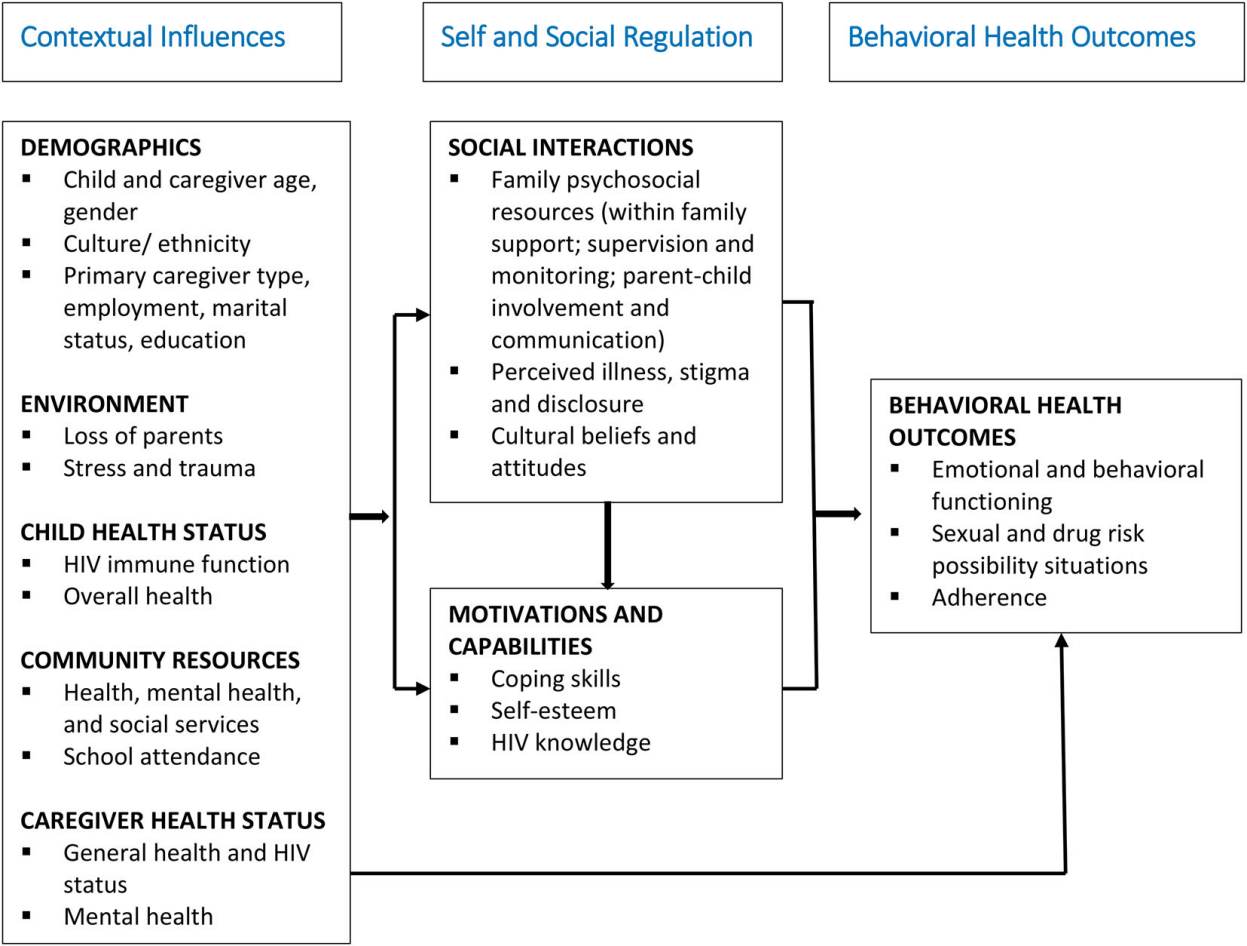
Modified SAT.

## Methods

This study uses baseline data pooled from the baseline interviews of a pilot randomized controlled trial (RCT) and the larger RCT for the VUKA Family Program, both based in KwaZulu-Natal, SA. VUKA (“Let’s Wake Up” in isiZulu) is a cartoon-based family intervention aimed at reducing risk behaviors in PHIV+ youth in poverty-impacted areas and is based on an evidence-based family mental health and prevention program for uninfected youth, Collaborative HIV and Adolescent Mental Health Program (CHAMP), that has been tested in multiple RCTs, including one set in SA (Bell et al., 2008; Bhana, McKay, Mellins, Petersen & Bell, 2010). The development of the VUKA intervention and the pilot trial have been described in previous publications (Bhana et al., 2014; Mellins et al., 2014). For both the pilot and the larger RCT that is ongoing, participants completed an extensive baseline interview with variables on mental health and the SAT constructs that are the basis for this study.

### Participants

Data from all participants who consented to be in the VUKA project and completed a baseline interview were included in these analyses. The VUKA pilot trial, conducted between 2008 and 2010, recruited participants from pediatric HIV clinics at two hospitals, one in Durban and one located near Durban in an urban area of KwaZulu-Natal. In the larger ongoing VUKA trial, initiated in 2012, participants were recruited from two large public hospitals located in Durban. Both the pilot and larger trial used the same recruitment procedures. Caregivers were referred to the study by medical staff and posters were placed in waiting areas for caregivers interested in enrolling. Staff also made announcements.

#### Inclusion criteria for both studies

To be eligible for VUKA, which targets younger adolescents (before most risk behaviors are initiated), youth had to meet the following criteria: (a) ages 9–14 years, (b) PHIV+, (c) aware of their HIV diagnosis (VUKA is a group-based intervention in which HIV is discussed), and (d) isiZulu speaking (the vast majority of patients in KwaZulu-Natal speak isiZulu). Caregivers included biological parents, guardians, relatives, and adoptive parents who had primary responsibility for the care of the youth and were isiZulu speaking.

#### Current sample

The pooled sample for these analyses contained 177 caregiver–child dyads who completed baseline interviews, 66 from the initial pilot trial and 111 from the larger RCT of the VUKA Family Project intervention trial. As seen in Table 1, the two samples did not differ significantly from each other with the only difference being a higher number of individuals experiencing food insecurity in the pilot intervention compared to the larger intervention trial.

**Table 1.** Demographic characteristics.

Pilot sample (*N* = 66)^a^			VUKA sample (*N* = 111)^a^			P*
HIV+ adolescents			HIV+ adolescents			
Gender	*N*	%	Gender	*N*	%	.71
Male	33	51	Male	48	47	
Female	32	49	Female	55	53	
Age mean ± SD	11.57 ± 1.16		Age mean ± SD	11.75 ± 1.57		.40
Caregiver characteristics			Caregiver characteristics			
Live in own place			Live in own place			.18
Yes	38	59	Yes	49	47	
No	27	41	No	56	53	
Receiving a grant for child			Receiving a grant for child			.26
Yes	52	81	Yes	76	72	
No	12	19	No	29	28	
Household Income			Household Income			.92
At least one person has job	20	32	At least one person has job	33	32	
At least one person has pension	19	30	At least one person has pension	35	34	
At least one person has job +			At least one person has job +			
one person has pension	21	33	one person has pension	30	29	
No one has job or pension	3	5	No one has job or pension	6	6	
Gone without food in past month			Gone without food in past month			.00
More than six times	11	17	More than six times	1	1	
4–6 times	10	16	4–6 times	2	2	
2–3 times	12	19	2–3 times	14	13	
One time	5	8	One time	2	2	
Never	25	40	Never	86	82	
Current regular employment			Current Regular employment			.06
Yes	19	30	Yes	17	16	
No	45	70	No	88	84	
Education			Education			.22
Eighth grade or less	22	35	<than eighth grade	26	25	
greater than eighth grade	41	65	>than eighth grade	79	75	
Caregiver HIV status			Caregiver HIV status			.33
Positive	39	74	Positive	65	64	
Negative	14	26	Negative	36	36	

^a^Valid sample size for each variable varies due to missingness.

*P-value: *t*-test for continuous variables and Chi-squared test for categorical variables (with continuity correction for 1 degree of freedom test).

### Procedures

In both studies, eligible, recruited participants were contacted by study staff, who described study procedures, and invited them to return for the first study assessment, if interested. Caregivers provided informed, written consent for themselves and their child; children provided written assent. Families were only enrolled if both consent and assent were obtained. For the baseline assessment, caregivers and adolescents were interviewed individually or in groups of 2–3 individuals (pilot only) with interviews lasting approximately one hour. The same procedures and selection criteria were used at baseline in the pilot and the larger study. The adults and adolescents were assessed separately from one another to ensure privacy. All responses were self-reported and recorded on paper (for the pilot study) or electronic tablets (for the larger trial). Participants were reimbursed for transportation and provided with a meal at each interview. Institutional Review Board approvals were obtained from all participating SA and US institutions.

### Measures

#### Youth behavioral outcomes

*Prosocial strengths and total difficulties* were assessed with the parent version of the Strengths and Difficulties Questionnaire (SDQ) (Goodman, 1997; Goodman, 2001). This 25-item scale is a well-validated and globally used measure of emotional and behavioral functioning that has been translated into multiple languages, including isiZulu (Youth in Mind, http://www.sdqinfo.com). It includes four subscales (emotional problems, conduct problems, hyperactivity, peer-relationship problems) that are used to calculate a total difficulty score, as well as a prosocial behavior score. Previous studies have found more than adequate psychometric properties, including validity, internal consistency, and tests–retest reliability (Goodman, 2001). (Cronbach’s *α* = 0.560 and 0.714 for prosocial strengths and total difficulties respectively in the combined sample.)

*Child depression* was measured using the short form of the Children’s Depression Inventory (CDI), a widely used measure with excellent reported psychometrics (Kovacs, 1985). The CDI includes 10-items, each rated on the degree of depression symptomatology experienced in the last two weeks (0–2). A total score is used. (Cronbach’s *α* = 0.299 in the combined sample.)

#### Contextual factors

##### Caregiver HIV status was determined by self-report

*Caregiver depression* was measured using the Beck Depression Inventory II (BDI-II; Beck, Brown, & Steer, 1996) in the VUKA pilot and the Center for Epidemiologic Studies Depression (CES-D) scale (Radloff, 1977) in the larger intervention trial. For the present analysis, clinically recommended cut-off scores for each measure were used to indicate if the caregiver showed positive symptoms of depression (depressed or not depressed) on whichever scale they completed.

##### BDI-II

Caregivers in the pilot study completed the 21-item BDI-II, rating characteristics and symptoms of depression. The measure has been used in locations and languages around the world, including SA, with excellent internal consistency (Beck et al., 1996; Steele & Edwards, 2008). (Cronbach’s *α* = 0.846 in the pilot sample).

##### CES-D

Caregivers in the larger intervention trial completed the 20-item CES-D Scale, which contains questions about depressive symptomatology in the past week. The CES-D Scale has shown strong psychometric properties in the USA and in SA, where it has been used with various populations, including HIV-infected adults, and has been translated into isiXhosa, Afrikaans, and isiZulu (Kuo & Operario, 2011; Myer et al., 2008). A cutoff score of 16 was used on this measure of depression. (Cronbach’s *α* = 0.894 in the larger trial.)

#### Social regulation factors

*Caregiver supervision* was measured using an instrument tapping caregiver supervision and monitoring created for previous trials of CHAMP in the US and SA with HIV− youth (Bell et al., 2008; McKay & Paikoff, 2007) that was completed by both caregivers and children. The caregiver and child versions include 17 and 14 items, respectively, which address rules, how parents keep track of their child’s whereabouts, and how often children are left in charge of the home. Inter-item reliabilities of the subscales in a sample of 500 urban parents of pre and young adolescent males ranged from .68 (rules) to .81 (extent of involvement/supervision) (Bell et al., 2008). (Cronbach’s *α* = 0.742 in the combined sample.)

*Youth and caregiver frequency and comfort* in communication was measured with two scales (comfort and frequency) each of which includes seven topics that caregivers and children often find hard to discuss, including drugs/alcohol, puberty, sex, STDs, and HIV/AIDS. Respondents rate the frequency of discussion about each topic and their level of comfort discussing each topic. The measure has been successfully used in previous CHAMP programs with good psychometric properties (Bell et al., 2008; Bhana et al., 2014). (Cronbach’s *α* = 0.771–0.815 in the combined sample.)

*Stigma* was examined as caregivers and children completed a measure of both externalized and internalized stigma related to HIV and AIDS that was originally developed for pediatric epilepsy (Westbrook, Bauman, & Shinnar, 1992) and used in the US version of CHAMP+ (McKay & Paikoff, 2007). It consists of eight items related to feelings about HIV and perceptions about how HIV affects social relationships. (Cronbach’s *α* = 0.472–0.869 in the combined sample.)

#### Self-regulation factors

*Self-concept* was measured with the short form of the child version of the Tennessee Self Concept Scale (TSCS-2), Second Edition (Fitts & Warren, 1996), which assesses components of self-esteem (identity, satisfaction, and behavior) over 6 specific domains of self-concept (physical, moral, personal, family, social, and academic) using 20 items. Good psychometric properties have been found in studies in SA and Uganda (Ssewamala, Han, & Neilands, 2009). (Cronbach’s *α* = 0.735 in the combined sample.)

*Coping* was only assessed in the pilot study using the Kidcope, a 15-item self-report measure (Spirito, Stark, & Williams, 1988). These items examine 10 subcategories indicating whether problem-solving, distraction, social support, social withdrawal, cognitive restructuring, self-criticism, blaming others, emotional expression, wishful thinking, or resignation mechanism was used. Each coping category refers to a primary description of a way of coping.

## Analysis

First, we used descriptive statistics to describe the sample characteristics of caregivers and PHIV+ adolescents. Differences between the two samples were statistically established using *t*-tests for continuous variables and Chi-squared tests for categorical variables, with a continuity correction for 1-degree of freedom test. Next, we used a simple pooling method to combine the two datasets and constructed a simple linear regression analysis to examine the bivariate association between the participants’ SDQ scores (which measured total difficulties and prosocial strengths) and contextual, social regulation and self-regulation factors in the modified SAT model (See Fig. 1). Additional adjusted analyses were also conducted. The independent variables in the adjusted model included one factor of interest, age, gender, and study indicator (pilot intervention versus larger VUKA intervention trial) to adjust for potential confounding. We then used CDI scores as the dependent variable and repeated all of the above bivariate and adjusted analyses. We reported the unadjusted and adjusted regression coefficient (*β*) for each factor in the regression model which represents the magnitude change of the outcome variable by one unit increase of the factor under consideration (unconditionally and conditionally, respectively). We declared the findings as statistically significant if the corresponding *p*-values were ≤.05. All statistical analyses were conducted using IBM SPSS Statistics 23.

## Results

### Participants

In the combined sample at baseline, the mean child age was 11.68 years (SD = 1.42) across studies; 56% lived with their mother, while others lived with fathers, siblings, or extended family members. The average caregiver age was 40 years (SD = 13.75) and 91% of caregivers were female. Table 1 presents the demographic composition of the participants from the pilot and larger trial separately and as noted previously, there were minimal differences.

Findings from the regression analysis suggested that factors from each level of influence in the SAT model are significantly associated with child resilience outcomes.

#### Total difficulties score

In the bivariate analysis, we found that lower total difficulties scores among children were associated with lower caregiver depression (*β* = 3.851, *p* < .001), less frequent caregiver-reported communication about difficult topics (*β* = 1.871, *p* = .007), more caregiver-reported supervision (*β* = −0.198, *p* = .041), and higher youth self-concept scores (*β* = −0.133, *p* = .006). These associations remained significant in the same direction after adjusting for age, gender, and study indicator (caregiver depression (*β* = 3.906, *p* < .001), caregiver-reported communication (*β* = 1.882, *p* = .009), and youth self-concept scores (*β* = −0.119, *p* = .020)), with the exception of caregiver supervision, which became non-significant (see Table 2).

**Table 2.** Factors associated with total difficulties score (SDQ).

Variable	Unstandardized *β*		Standardized *β*		*t*-score		*p*-value	
** **	Unadj.^a^	Adj.^b^	Unadj^a^	Adj.^b^	Unadj^a^	Adj.^b^	Unadj^a^	Adj.^b^
*Contextual influences*								
Caregiver depressed	3.851	3.906	0.338	0.341	4.377	4.269	<0.001	<0.001
*Social regulation*								
Caregiver-reported communication frequency	1.871	1.882	0.220	0.222	2.753	2.658	0.007	0.009
Caregiver-reported supervision total score	−0.198	−0.155	−0.166	−0.122	−2.058	−1.365	0.041	0.174
*Self-regulation*								
Self-concept score (child reported)	−0.133	−0.119	−0.228	−0.205	−2.784	−2.363	0.006	0.020

^a^Unadjusted results represented the findings from simple linear regression analysis in which only one independent variable was included in the model.

^b^Adjusted results were obtained from multiple linear regression analysis. In each adjusted model, independent variables included one factor of interest and three potential confounders (i.e., age, gender, and study indicator) to adjust for potential confounding.

#### Prosocial strengths score

As shown in Table 3, simple linear regression analysis revealed that greater prosocial strengths were associated with greater caregiver-reported frequency of communication with children about difficult topics (*β* = 0.649, *p* < .001) and communication comfort (*β* = 0.437, *p* = .037), as well as youth use of wishful thinking as a coping method (*β* = 5.390, *p* = .009). After adjusting for age, gender, and study indicator, greater frequency of communication about difficult topics (*β* = 0.722, *p* = .020) and use of wishful thinking (*β* = 5.532, *p* = .009) were still significantly associated with stronger prosocial strengths; however, communication comfort just fell short of significance (*β* = 0.417, *p* = .064).

**Table 3.** Factors associated with Prosocial strengths score (SDQ).

Variable	Unstandardized *β*		Standardized *β*		*t*-score		*p*-value	
** **	Unadj.^a^	Adj.^b^	Unadj^a^	Adj.^b^	Unadj^a^	Adj.^b^	Unadj^a^	Adj.^b^
*Contextual factors*								
Receive any grants for children	0.638	0.552	0.151	0.130	1.943	1.572	0.054	0.118
*Social regulation*								
Caregiver-reported communication frequency	0.649	0.722	0.251	0.278	3.284	3.472	0.001	0.020
Caregiver-reported communication comfort	0.437	0.417	0.165	0.155	2.105	1.867	0.037	0.064
*Self-regulation*								
Wishful thinking coping mechanism (pilot only)	5.390	5.532	0.332	0.343	2.684	2.690	0.009	0.009

^a^Unadjusted results represented the findings from simple linear regression analysis in which only one independent variable was included in the model.

^b^Adjusted results were obtained from multiple linear regression analysis. In each adjusted model, independent variables included one factor of interest and three potential confounders (i.e., age, gender, and study indicator) to adjust for potential confounding.

#### CDI score

The unadjusted findings suggested that lower level of depression in children was associated with higher caregiver education (*β* = −0.412, *p* = .010), lower household density (*β* = 0.092, *p* = .041), and greater food security (i.e., less reported hunger in the past month) (*β* = 0.254, *p* = .029). Social regulation factors were also associated with depression in children. Lower levels of youth-reported supervision by caregiver (*β* = −1.667, *p* < .001) and less likelihood of youth seeking social support (*β* = −0.429, *p* = .003) were associated with higher levels of youth depression. Within self-regulation factors, higher self-concept scores (*β* = −0.076, *p* < .001) and lower levels of internal stigma (*β* = 0.655, *p* = .027) were associated with lower levels of youth depression, while the use of social withdrawal (*β* = 1.297, *p* = .022) and resignation (*β* = 1.156, *p* = .036) as coping methods were associated with higher levels of depression. Adjusting for age, gender and study indicator, caregiver education (*β* = −0.399, *p* = .010), as well as the social regulation factors (caregiver supervision (*β* = −1.261, *p* = .012) and likelihood of seeking social support (*β* = −0.453, *p* = .002)) and self-regulation factors (self-concept scores (*β* = −0.067, *p* < .001), internal stigma (*β* = 0.608, *p* = .040), and use of resignation as coping method (*β* = 1.152, *p* = .041)) still remained significantly associated with child depression in the same direction as unadjusted findings (see Table 4).

**Table 4.** Factors associated with CDI score.

Variable	Unstandardized *β*		Standardized *β*		*t*-score		*p*-value	
	Unadj.^a^	Adj.^b^	Unadj ^a^	Adj.^b^	Unadj ^a^	Adj.^b^	Unadj^a^	Adj.^b^
*Contextual factors*								
Caregiver education	−0.412	−0.399	−0.204	−0.198	−2.611	−2.603	0.010	0.010
How many people share house?	0.092	0.081	0.162	0.144	2.065	1.849	0.041	0.066
In the past month, how often have you or your family gone without enough food to eat?	0.254	0.074	0.174	0.051	2.202	0.573	0.029	0.568
*Social regulation*								
Youth-−reported supervision	−1.667	−1.261	−0.336	−0.254	−4.550	−2.554	<0.001	0.012
Youth likelihood of seeking support	−0.429	−0.453	−0.231	−0.244	−3.009	−3.177	0.003	0.002
*Self-regulation*								
Tennessee self-concept score	−0.076	−0.067	−0.389	−0.343	−5.414	−4.624	<0.001	<0.001
Youth-reported internal stigma	0.655	0.608	0.173	0.162	2.226	2.070	0.027	0.040
Social withdrawal coping mechanism (pilot only)	1.297	1.068	0.293	0.241	2.356	1.863	0.022	0.068
Resignation coping mechanism (pilot only)	1.156	1.152	0.262	0.261	2.138	2.084	0.036	0.041

^a^Unadjusted results represented the findings from simple linear regression analysis in which only one independent variable was included in the model.

^b^Adjusted results were obtained from multiple linear regression analysis. In each adjusted model, independent variables included one factor of interest and three potential confounders (i.e., age, gender, and study indicator) to adjust for potential confounding.

## Discussion

This is one of the few studies examining theoretically informed (SAT) correlates of mental health outcomes in PHIV+ early adolescents growing up in a low- to middle-income country. With the advent of antiretroviral treatment, concerns about child and adolescent survival has naturally shifted to the well-being and social implications of having a stigmatizing and transmittable disease (Lowenthal et al., 2014). In this context, while a number of studies have reported on the negative consequences associated with PHIV+ status (Mellins & Malee, 2013; Mellins et al., 2009; Mellins et al., 2012), it is just as important to examine those elements that contribute to the positive development of PHIV+ youth (Mellins et al., 2014; Sherr, Croome, Parra Castaneda, & Bradshaw, 2014).

Using the results from baseline data of two RCTs of the VUKA family intervention, the study set out to examine the contextual, social, and self-regulation factors that are associated with positive mental health outcomes among PHIV+ youth. The data indicated that there are factors from each of three SAT domains that were associated with youth prosocial behaviors, total difficulties and depression, providing guidance for much-needed evidence-based interventions for PHIV+ children reaching adolescence, worldwide.

Contextual factors appeared to have a significant influence on PHIV+ adolescent resilience, including household size and caregiver factors. Household size may reflect caregiver burden arising from a crowded household as well as emotional and financial burden (DSD, SASSA, & UNICEF, 2012). Conversely, as has been found in other studies of vulnerable youth affected by HIV, caregiver factors, such as better education and more limited depression symptoms may increase caregiver capacity to provide emotional support to PHIV+ adolescents (Cluver, Boyes, et al., 2013; Cluver, Orkin, et al., 2013).

Social regulation factors within family contexts were also associated with positive youth outcomes particularly the frequency with which caregivers communicated and provided supervision for their children. Also, greater supervision by caregivers was inversely associated with mental health (lower depression) among youth. Social regulation factors that include parental warmth and care and supervision by a caregiver may prove to be vital to promoting resilience among youth (Fergus & Zimmerman, 2005). However, less communication about difficult topics was associated with fewer total behavioral problems. It is possible that youth who have fewer behavioral difficulties may require less discussion about problems with their caregivers.

Finally, self-regulation is closely related to achieving healthy cognitive, behavioral, emotional, and mental health functioning (National Research Council and Institute of Medicine, 2009) and has implications for how PHIV+ adolescents manage their chronic illness. The coping methods employed by PHIV+ adolescents included resignation and wishful thinking. While these avoidant coping strategies may not be viewed as health-promoting in themselves, as they are reactive and lack active problem-solving components, they may be appropriate for some youth in a limited resource context filled with stressors out of the control of children. It is also possible that they may later become the basis of youth developing more active coping responses. It is also likely a feature of the developmental status of early adolescents in this study. Actively seeking support from others, higher self-concept, and less internalized stigma were all correlated with better mental health among PHIV+ adolescents. The development of a healthy sense of self (self-concept) and greater acceptance of their HIV status has been found to be important in other vulnerable youth (Ungar et al., 2007) and thus, may also be important to promoting resilience in PHIV+ adolescents.

The study has a number of limitations that affect the generalizability of our findings. First, even though the original samples were randomized, they do represent a sample of convenience for the purposes of this paper. It is likely that given that we drew our samples from those in care and who consented to participate in the RCTs, it may not reflect the larger PHIV+ adolescent population. Also, the data are cross-sectional and consists of self-report data which further limits the conclusions that can be drawn. Nor is it possible to disentangle the factor of time between the pilot and the larger trial. Finally, in an effort to minimize discomfort to respondents, we endeavored to keep our measurement battery to less than an hour and many other important factors in understanding PHIV+ adolescent resilience may not have been assessed.

## Conclusion

In spite of the limitations, this study highlights the importance of studying high-risk populations, particularly in ways that avoid promoting only deficit-focused perspectives on PHIV+ adolescents. Despite the context of poverty and few resources, this study suggests that the mechanisms with which PHIV+ adolescents and their caregivers adapt and respond to a chronic illness are as much a function of the familial and community resources to which they have access as it is to the availability of early treatment. Participating families demonstrate resilience in various ways which appear to be nested within various contextual and social arrangements. Although longitudinal studies with larger samples will be critical to determine pathways of causality, these findings are encouraging and align with existing findings from other vulnerable populations, namely that parental supervision and communication, and youth self-esteem and low internalized stigma are factors that may promote resilience in PHIV+ youth. Thus, our data provide an important base for much-needed evidence-based interventions that are health- and mental health-promoting among PHIV+ youth and their caregivers.
